# High expression of FUNDC1 predicts poor prognostic outcomes and is a promising target to improve chemoradiotherapy effects in patients with cervical cancer

**DOI:** 10.1002/cam4.1112

**Published:** 2017-07-18

**Authors:** Hailing Hou, Puchun Er, Jingjing Cheng, Xiuli Chen, Xiaofeng Ding, Yuwen Wang, Xi Chen, Zhiyong Yuan, Qingsong Pang, Ping Wang, Dong Qian

**Affiliations:** ^1^ Key Laboratory of Cancer Prevention and Therapy Department of Radiotherapy Tianjin Medical University Cancer Institute and Hospital National Clinical Research Center for Cancer Tianjin's Clinical Research Center for Cancer Tianjin China

**Keywords:** Apoptosis, cervical cancer, chemoradiotherapy, FUNDC1, prognostic biomarker

## Abstract

FUN14 domain containing 1 (FUNDC1) is an important molecule in receptor‐dependent mitophagy. However, the roles of FUNDC1 in human cancer biology remain unknown. The aim of this study was to explore the expression and roles of FUNDC1 in cervical cancer. Immunohistochemistry and Western blotting were applied to detect the expression of FUNDC1, and small‐hairpin RNA was applied to inhibit the expression of endogenous FUNDC1 in cervical cancer cells. MTT assays and Flow cytometric analysis were applied to examine cell proliferation and apoptosis. Immunofluorescence was used to detect the formation of γH2AX foci and evaluate the extent of DNA damage. Compared with corresponding adjacent noncancerous cervical tissues, the expression of FUNDC1 in cervical cancer cells was significantly increased. High expression of FUNDC1 and the prognosis of patients with cervical cancer were correlated negatively, which could be used as an independent prognostic factor for overall survival and disease‐free survival. Depletion of FUNDC1 significantly inhibited the proliferation of tumor cells, induced apoptosis, and enhanced cell sensitivity to cisplatin and ionizing radiation (IR). Our data suggested that FUNDC1 can be used as a prognostic biomarker in patients with cervical cancer, and may be a new therapeutic target to improve the antitumor effects of chemoradiotherapy.

## Introduction

Cervical cancer is a gynecological cancer with the second highest incidence in women. The main histological type is squamous cell carcinoma, accounting for more than 80% of cervical cancer. More than 85% of cervical cancer occurs in developing countries 1. There are about 130,000 new cases in China every year, and each year about 50,000 patients die of cervical cancer 2. Thus, cervical cancer is not only a global public health issue but also the leading cause of death related to cancers of the reproductive system in developing countries 1. Clinicians usually devise treatment strategies based on the FIGO (International Federation of Gynaecology and Obstetrics) staging. Recent studies show that lymph node metastasis, primary tumor size, and parametrial involvement indicators are prognostic factors of cervical cancer 3. However, the relevant known factors are insufficient to guide clinicians to select an individualized treatment plan and predict the effects. Several studies have shown that many genes, such as annexin A2, Sam68, and HDAC10 4, 5, 6, may be effective prognostic markers of cervical cancer, but subsequent studies are needed to confirm the significance of these new biomarkers in guiding treatment and predicting curative effects.

Mitochondria are specialized organelles with a closed bilayer lipid membrane in eukaryotic cells, which perform many cellular functions to maintain intracellular homeostasis. Mitochondrial health is essential for homeostasis of the whole body, and mitochondrial dysfunction is involved in the development of a variety of human diseases including cancer 7, 8. Mitochondria generate reactive oxygen species (ROS) in the process of ATP production, and ROS can induce oxidative damage of mitochondrion‐associated proteins, resulting in mitochondrial damage. Damaged mitochondria can promote activation of the apoptotic pathway by the release of cytochrome C. Accumulation of mitochondrial damage is not conducive to cell survival, and can lead to various diseases. The body removes excessively damaged mitochondria through a variety of pathways to maintain homeostasis. The main mechanism is mitophagy. There are two categories of mitophagy: ubiquitin pathway‐mediated mitophagy and receptor‐dependent mitophagy 8. FUN14 domain containing 1 (FUNDC1) is an important molecule in newly discovered receptor‐dependent mitophagy. As a mitochondrial outer‐membrane protein, FUNDC1 has three transmembrane regions. Its N‐terminal is located within cytoplasm, whereas the C‐terminal is located between the inner and outer membranes of mitochondria. Studies show that, under hypoxic conditions, mitochondrial phosphoglyceromutase 5 (PGAM5) dephosphorylates FUNDC1, facilitating its combination with light chain 3 (LC3), which results in selective autophagy fusion and clearance of damaged mitochondria through autophagy 9. In addition, FUNDC1 regulates mitochondrial fission and fusion through dynamin‐related protein 1 (DRP1), maintains homeostasis, and participates in the programmed cell necroptosis regulated by the RIP3/MLKL pathway 10, 11.

Many mitophagy pathway proteins, such as Parkin, BNIP (BCL2/adenovirus E1B 19 kd‐interacting protein), and NIX, and other proteins have abnormal expression in tumors and may be related to the prognosis and treatment of patients 12. However, the role of FUNDC1 in tumors remains unclear. Therefore, this study aimed to explore the relationship between the expression of FUNDC1 and the prognosis of patients with cervical cancer. We also investigated the possible biological mechanisms of FUNDC1 in the sensitivity of cervical cancer cells to chemoradiotherapy.

## Materials and Methods

### Tissue specimens and cell cultures

A total of 82 patients with early‐stage cervical cancer (FIGO stage IB–IIA) who underwent radical hysterectomy and lymphadenectomy surgery followed by adjuvant platinum‐based chemoradiotherapy between 2010 and 2013 at Tianjin Medical University Cancer Institute and Hospital (Tianjin, China) were enrolled in this study. All tissue samples were obtained with informed consent under Institutional Review Board–approved protocols. Clinical stage was assessed according to the revised FIGO staging for carcinoma of the cervix 13. Tumor pathological type and differentiation grade were defined according to World Health Organization criteria 14. The clinicopathological characteristics of the patients studied were summarized in Table**** 1. A total of 82 primary tumor samples were obtained with informed consent under Institutional Review Board–approved protocols. Thirty‐five of these 82 patients also have correspondence paired normal cervical tissue. All paraffin‐embedded samples were collected at the Tianjin Medical University Cancer Institute and Hospital (Tianjin, China). The cervical cancer cases selected were based on clear pathological diagnosis and follow‐up data. All samples were formalin fixed, paraffin embedded, and pathologically diagnosed. This study was approved by the Institute Research Ethics Committee of Tianjin Medical University Cancer Institute and Hospital.

**Table 1:** Clinicopathological characteristics and FUNDC1 expression in the cervical cancer patients

Characteristics	FUNDC1		*P* value
	Low expression, *N* (%)	High expression, *N* (%)	
Age, years			0.39
<48	13 (15.9)	20 (24.4)	
≥48	24 (29.3)	25 (30.5)	
FIGO stage			0.56
IA2	1 (1.2)	1 (1.2)	
IB1	12 (14.6)	9 (11.0)	
IB2	1 (1.2)	5 (6.1)	
IIA	19 (22.4)	22 (26.8)	
IIB	4 (4.7)	8 (9.8)	
Tumor size, cm			0.13
<4	34 (41.5)	36 (43.9)	
≥4	3 (3.7)	9 (10.8)	
Histology			0.54
SCC	34 (41.5)	41 (50.0)	
AC	2 (2.4)	1 (1.2)	
ASC	1 (1.2)	3 (3.7)	
Histological differentiation			0.73
Well	2 (2.4)	1 (1.2)	
Moderate	28 (34.1)	36 (43.9)	
Poor	7 (8.5)	8 (9.8)	
Deep stromal invasion			0.73
No	4 (4.9)	6 (7.3)	
Yes	33 (40.2)	39 (47.6)	
Lymphovascular space involvement			0.98
No	32 (39.0)	39 (47.6)	
Yes	5 (6.1)	6 (7.3)	
Positive parametrium			0.37
No	33 (40.2)	37 (45.1)	
Yes	4 (4.9)	8 (9.8)	
Positive surgical margin			0.45
No	35 (42.7)	44 (53.7)	
Yes	2 (2.4)	1 (1.2)	
LNM			0.39
No	25 (30.5)	25 (30.5)	
Yes	6 (7.3)	13 (15.9)	
Chemotherapy			0.68
No	1 (1.2)	2 (2.4)	
Yes	36 (43.9)	43 (52.4)	
Radiotherapy			1
No	0 (0.0)	0 (0.0)	
Yes	37 (45.1)	45 (54.9)	

SCC, squamous cell carcinoma; AC, adenocarcinoma; ASC, adenosquamous carcinoma; LNM, lymph node metastasis.

The cervical cancer cell lines (Hela229 and CaSKi) were obtained from ATCC (American Type Culture Collection). Cells were cultured <3 months after resuscitation and were maintained in RPMI 1640 media (Gibco Laboratories, Buffalo, Grand Island, NY) with 10% (vol/vol) fetal bovine serum (Gibco Laboratories) at 37°C in a 5% CO2 incubator. Cells were authenticated by short tandem repeat (STR) fingerprinting at the Medicine Lab of Forensic Medicine Department of Sun Yat‐sen University (Guangzhou, China).

### Tissue microarray

Tissue microarrays (TMAs) were constructed according to a previously described method 15. Triplicate 0.6‐mm‐diameter cylinders (two identical cylinders taken from intratumoural tissue and one cylinder from peritumoural tissue) were punched from representative areas of an individual donor tissue block, and reembedded into a recipient paraffin block in a defined position, using a tissue array instrument (Beecher Instruments, Silver Spring, Maryland).

### Immunohistochemistry

Paraffin‐embedded tissues were cut into 4 *μ*m sections and rehydrated through a graded alcohol series, subjected to heating for antigen retrieval, allowed to cool to room temperature, immersed in 3% hydrogen peroxide for 5–10 min, and then rinsed with phosphate‐buffered saline (PBS). The sections were incubated with normal goat serum to block nonspecific binding, followed by incubation overnight with primary antibodies at 4°C, and then rinsed with PBS. Subsequently, the sections were incubated with a secondary antibody for 15 min at 37°C, washed with PBS, and then stained with diaminobenzidine. Finally, the sections were counterstained with hematoxylin, dehydrated, and mounted.

### IHC evaluation

The expression of FUNDC1 was assessed by three independent pathologists (H. Hou, J. Cheng, and D. Qian) who were blinded to clinical follow‐up data. Their conclusions were in complete agreement for 85% of cases indicating this scoring method was highly reproducible. If two or all three agreed with the scoring results, the value was selected. If the results were completely different, then the pathologists worked collaboratively to confirm the score.

For evaluation of FUNDC1 staining, a semiquantitative scoring criterion was used, in which both staining intensity and positive areas were recorded. A staining index (values 0–16) obtained as the intensity of positive staining (week, 1; moderate low, 2; moderate high, 3; strong, 4) and the proportion of immune‐positive cells of interest (0%, 0; <10%, 1; 10–50%, 2; 51–80%, 3; >80%, 4) were calculated. Finally, cases were classified into two different groups: low‐expression cases (score 0–8) and cases with high expression (score 8–16).

### RNA extraction, reverse transcription, and real‐time PCR

Data S1.

### Plasmid construction, lentivirus production, and transduction

Plasmid containing the validated shRNAs targeted FUNDC1 was cloned into the vectors pLLU2G (kindly provide by professor Dan Xie, Cancer Center, Sun Yet‐Sen University) used in this study are derived from pLL3.7 and contain separate GFP and short‐hairpin RNA (shRNA) expression elements as well as required for lentiviral packaging 42. The target sequences of FUNDC1 for constructing lentiviral shRNA are 5′‐ TTAAGAAACGAGCGAACAA ‐3′ (shFUNDC1#1) and 5′‐ AAGTGATGACGACTCTTAT ‐3′ (shFUNDC1#2). For rescue experiments, a FUNDC1 construct (mutations underlined: TTGAGGAATGAACGCACCA) resistant to the siRNA used (TTAAGAAACGAGCGAACAA) was cloned into a pCDH cDNA expression lentivector (System Biosciences, Mountain View, CA). The mutations do not affect the FUNDC1 protein sequence. Then the lentiviral expression constructs (pCDH‐ FUNDC1) and packaging plasmids mix were cotransfected into 293 cells to generate the recombinant lentivirus according to the manual.

### Western blotting assay

Cells were lysed in lysis buffer. BCA kit (Pierce, Rockford, IL) was used to determine protein concentrations. SDS‐PAGE and Western blotting were done according to standard procedures. Proteins were detected with antibodies recognizing FUNDC1, BCL2L1 (BCL‐XL), LC3, and Caspase‐3 (Abcam, Cambridge, MA). GAPDH (Santa Cruz Biotechnology, California, CA) was used as loading control.

### MTT proliferation assay

Cell viability was measured with the use of 3‐(4, 5‐dimethylthiazol‐2‐yl)‐2, 5‐diphenyl tetrazolium bromide (MTT) proliferation assay (Sigma, St Louis, MO). Briefly, cells were seeded in 96‐well plates and cultured. Cell viability was examined following the standard procedures. Experiments were done in triplicate.

### Annexin V‐APC/PI flow cytometry apoptosis assay

Annexin V‐APC and propidium iodide (PI) stains were used to determine the percentage of cells undergoing apoptosis. The apoptosis assay was conducted using the protocol supplied by the manufacturer (BD Biosciences, Bedford, MA). Each sample was then subjected to analyses by flow cytometry (BD FACSCanto II Flow Cytometer, BD Biosciences).

### Clonogenic survival assay

Survival following radiation exposure was defined as the ability of the cells to maintain their clonogenic capacity and to form colonies. Hela229 and CaSKi cells were counted and seeded for colony formation in six‐well plates with 50–5000 cells per well. After incubation intervals of 14–21 days, colonies were stained with crystal violet and manually counted. Colonies consisting of 50 cells or more were scored, and five replicate wells containing 10–150 colonies per well were counted for each treatment.

### Immunofluorescence

Cells grown on coverslips were fixed in 4% paraformaldehyde. Fixed cells were permeabilized with 1% Triton‐100 and blocked with 10% normal goat serum. Cells were then incubated with primary antibody for 2 h at 37° in a humid chamber and were than incubated with the secondary antibodies for 1 h. Immunofluorescence images were captured using with FV10‐ASW viewer software (Olympus, Tokyo, Japan). Primary antibodies used were γH2AX (Cell Signaling, Danvers, MA), FUNDC1, and LC3 (Abcam, Cambridge, MA).

### Statistical analysis

Statistical analysis was carried out using SPSS 17.0 (SPSS Standard version 17.0, SPSS Inc., Chicago, IL). Student's *t*‐test was used to analyze the results expressed as mean ± SD. The Chi‐squared test or Fisher's exact test was used to analyze the association of FUNDC1 expression and clinical‐pathological parameters. The survival curves were plotted by Kaplan–Meier analysis. Differences were considered significant when the *P* < 0.05.

## Results

### Significantly high FUNDC1 expression occurs in cervical cancer tissue

**Figure 1:**
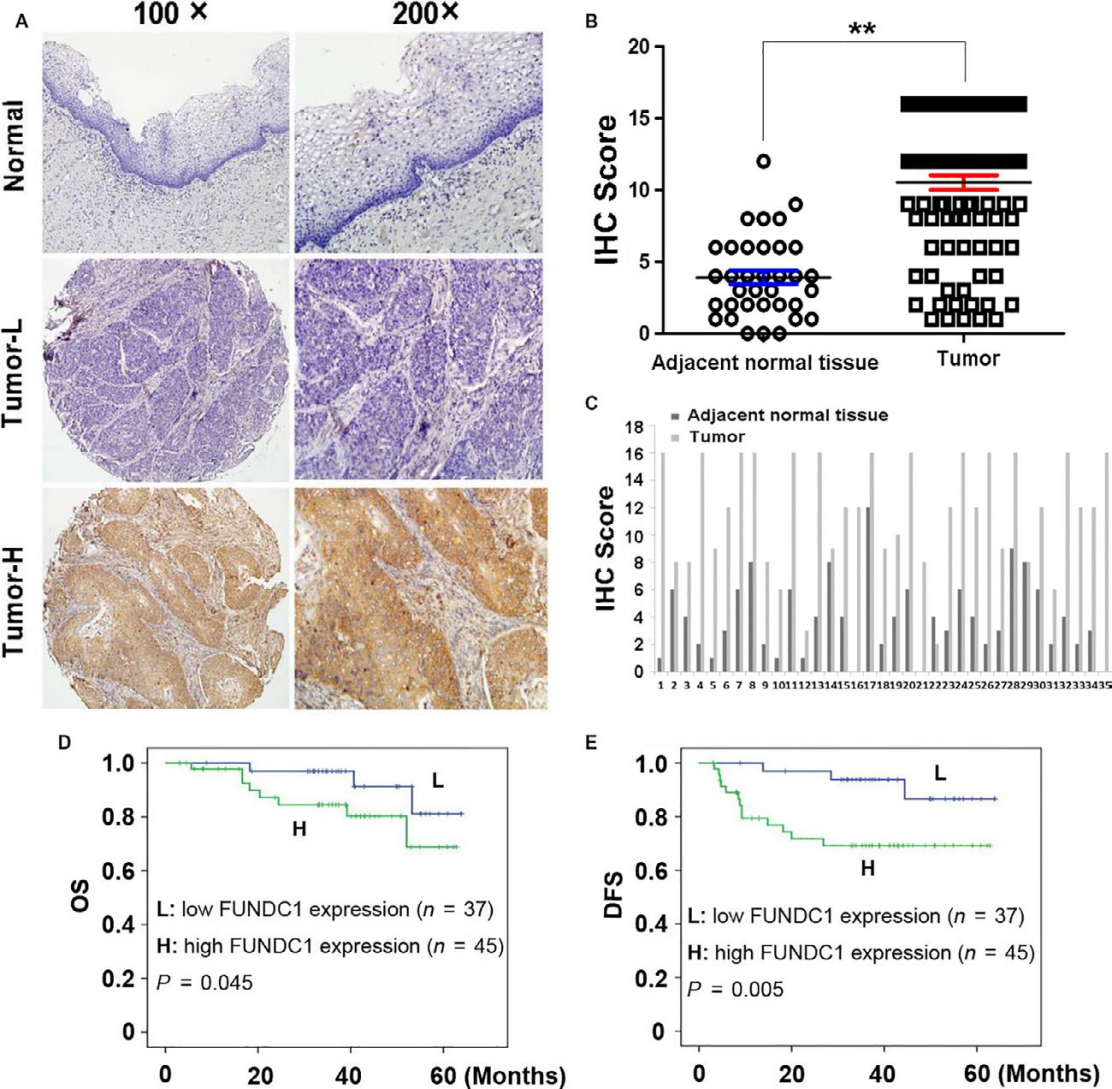
FUNDC1 expression in cervical cancer tissues and its prognostic significance in patients. (A) Normal: A correspondence normal cervical tissue (Case 12) shows negative expression of FUNDC1 (IHC score: 0). Tumor‐L: A cervical cancer tissue (Case 37) exhibited low expression of FUNDC1 (IHC score: 2). Tumor‐H: A cervical cancer tissue (Case 5) exhibited high expression of FUNDC1 (IHC score: 12). (B) Statistical analysis revealed significantly higher expression of FUNDC1 in cervical cancer tissues (**P <* *0.01, Student's *t*‐test). (C) The expression of FUNDC1 in 35 cervical cancer samples which have paired adjacent normal lung tissues. (D and E) Kaplan–Meier plots showed overall survival (D) and disease‐free survival (E) curves of our enrolled 82 patients, according to FUNDC1 expression levels in the primary tumor (L, low expression of FUNDC1; H, high expression of FUNDC1. Log‐rank test).

In this study, immunohistochemistry was used to detect FUNDC1 protein expression in paraffin‐embedded specimens of postoperative tumor tissues from 82 cervical cancer patients and in 35 samples of normal adjacent cervical tissue as the control (Fig. 1A). Among the 82 tumor tissue specimens, 45 cases (54.9%) had high FUNDC1 protein expression (score: > 8). FUNDC1 protein expression in the 35 samples of normal adjacent tissue was negative or low. Thus, the rate of high FUNDC1 protein expression in cervical cancer tissues was significantly higher than that in normal cervical tissues (Fig. 1B and C, **P <* *0.01). Furthermore, we used Real‐time PCR to measure the mRNA level of FUNDC1 in 12 paired cervical cancer (CC) and adjacent normal cervical tissues (ANTs). Compared with their ANTs, all of CCs had higher FUNDC1 mRNA expression (Fig. S1).

### FUNDC1 protein expression and the prognosis of patients with early‐stage cervical cancer are correlated negatively

We conducted statistical analysis of the differences in FUNDC1 protein expression in overall survival (OS) and disease‐free survival (DFS) of the 82 cervical cancer patients. Kaplan–Meier analysis was applied to assess prognosis, and the Log‐rank test was used to compare differences between high and low FUNDC1 expression groups. The median follow‐up period was 38 months for all patients, and 11 patients died of tumor‐related complications during this period (27.3% were low FUNDC1 expression and 72.7% were high FUNDC1 expression). Univariate analysis showed that FUNDC1 protein expression was negatively correlated with OS and DFS (Tables 2, 3). Multivariate statistical analysis showed that FUNDC1 expression was an independent prognostic factor to determine cervical cancer (OS: HR: 0.273, 95% CI: 0.071–1.058, *P* = 0.045; DFS, HR: 0.197, 95% CI: 0.056–0.694, *P* = 0.005, Table 2). Kaplan–Meier analysis showed that DFS and OS of patients with high FUNDC1 expression were significantly lower than those of patients with low FUNDC1 expression (Fig. 1D and E).

**Table 2:** Prognostic factors for OS and DFS: univariate and multivariate analyses

Factors	*P* ‐value			
	Univariate		Multivariate	
	OS	DFS	OS	DFS
Age	0.079	0.000	0.212	0.252
FIGO stage	0.001	0.006	0.001	0.006
Tumor size	0.698	0.208	0.698	0.208
Histology	0.029	0.232	0.030	0.232
Histological differentiation	0.758	0.879	0.758	0.879
Deep stromal invasion	0.155	0.349	0.155	0.350
Lymphovascular space involvement	0.563	0.563	0.564	0.564
Positive parametrium	0.001	0.003	0.001	0.004
Positive surgical margin	0.416	0.623	0.416	0.624
LNM	0.042	0.030	0.042	0.030
Chemotherapy	0.001	0.066	0.001	0.066
State of FUNDC1	0.045	0.005	0.046	0.005

OS, overall survival; DFS, disease‐free survival; LNM, lymph node metastasis; FUNDC1, FUN14 domain containing 1.

**Table 3:** Patients survival and FUNDC1 expression in the cervical cancer patients

	FUNDC1		*P* value
	Low expression	High expression	
5 years OS (%)	84	62.6	0.045
5 years DFS (%)	88.5	66.7	0.005
Median OS (month)	40.9	36.7	0.045
Median DFS (month)	38.9	33.8	0.005

### Depletion of FUNDC1 inhibits cervical cancer cell proliferation and induces apoptosis

**Figure 2:**
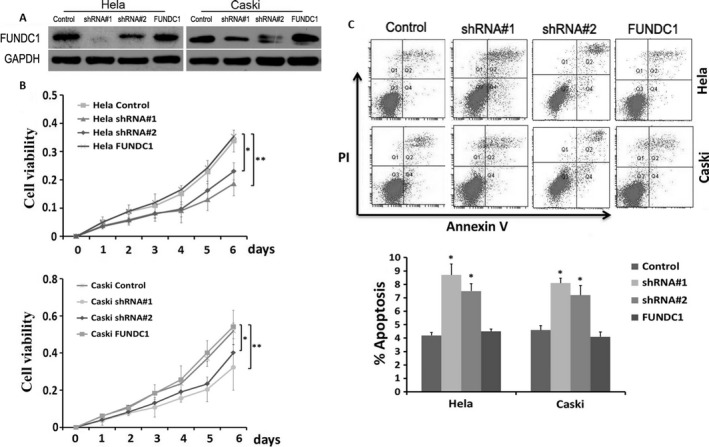
Inhibition of FUNDC1 suppresses tumor cell growth and promotes apoptosis of cervical cancer cells. (A) Two shRNAs targeting FUNDC1 mRNA (shFUNDC1#1 and shFUNDC1#2) were introduced into Hela229 and CaSKi cells for stable knockdown of FUNDC1. Then, pCDH‐FUNDC1 lentiviral particles were transduced into the above FUNDC1‐silenced cell lines to replenish FUNDC1 expression. The levels of FUNDC1 were detected by Western blotting. Expression was normalized against endogenous GAPDH levels. (B) Cell growth rate was suppressed by FUNDC1 knockdown in Hela229 (up) and CaSKi (down) cells detected by MTT assay. Results are expressed as mean ± SD of three independent experiments. (**P <* *0.01). (C) Depletion of FUNDC1 promotes apoptosis of cervical cancer cells. Cell apoptotic death events were monitored by Annexin V/PI staining and flow cytometry assays. The percentage of cell apoptosis was shown as the mean ± SD from three independent experiments. (*P <* *0.05). shRNA#1 had a better effect and was chosen for further study.

To explore the roles of FUNDC1 in the radiotherapy response of cervical cancer cells, we infected HeLa and Caski cells with lentivirus carrying FUNDC1 short‐hairpin RNA (shRNA#1 and shRNA#2) and the corresponding control Scramble shRNA (Control groups). The knockdown efficiency of FUNDC1 was confirmed by Western blotting (Fig. 2A), and shRNA#1 had a better silencing effect than shRNA#2. MTT assays were applied to examine cell proliferation. The results showed that, after inhibition of FUNDC1 expression, the proliferation of HeLa and Caski cells was inhibited significantly (Fig. 2B). In addition, we applied Annexin V‐APC/PI staining to detect apoptosis after inhibition of FUNDC1. Flow cytometric analysis showed that FUNDC1 gene silencing promoted the apoptosis of both cell lines (Fig. 2C). shRNA#1 was chosen for further study because of its better effect on cell proliferation and apoptosis than that of shRNA#2. To make sure that the levels of FUNDC1 modulate the viability and apoptosis of CC cells, we replenished the levels of FUNDC1 in FUNDC1‐silenced HeLa and Caski cells by infection with recombinant lentivirus encoding a FUNDC1 construct resistant to used short‐hairpin RNA (shRNA#1) as described in Materials and Methods (Fig. 2A). Our gain‐of‐function studies revealed that ectopic overexpression of FUNDC1 promoted cell proliferation and inhibited apoptosis in both FUNDC1‐silenced HeLa and Caski cells (Fig. 2B and C).

### Inhibition of FUNDC1 enhances the sensitivity of cervical cancer cells to cisplatin and ionizing radiation

**Figure 3:**
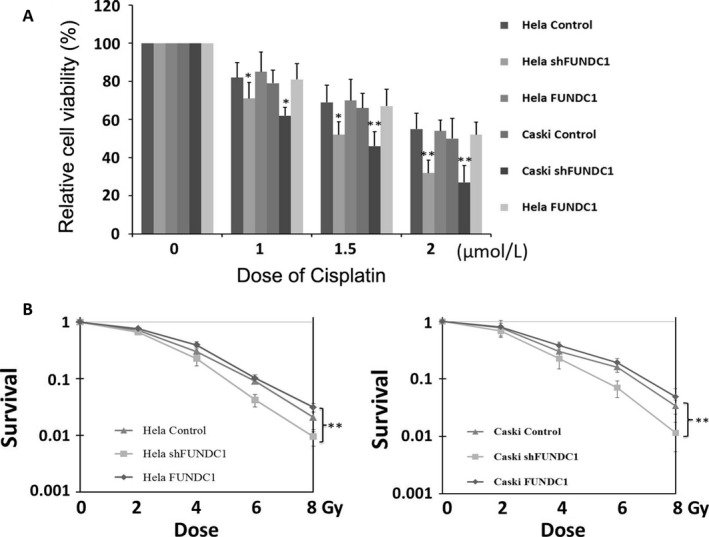
Silencing FUNDC1 enhances cervical cancer cells chemosensitivity to cisplatin and radiosensitivity in vitro. (A) FUNDC1 knockdown enhanced Hela229 and CaSKi cells sensitivity to cisplatin. Cells were treated with cisplatin for 24 h at the indicated concentration. The cell viabilities were detected by MTT assay. (B) Silencing of FUNDC1 enhanced radiosensitivity in Hela229 (left) and CaSKi (right) cells. The responses of cells to IR at indicated dose were examined by clonogenic survival assays. All data represent the mean ± SE derived from three individual experiments with triplicate wells. (*P <* *0.05, **P <* *0.01, Student's *t*‐test)

All postoperative cervical cancer patients in this study had accepted cisplatin‐based adjuvant chemotherapy and radiotherapy. Therefore, we investigated the effect of FUNDC1 on the sensitivity of HeLa and Caski cells to chemoradiotherapy. After 24 h of cisplatin treatment at various concentrations, MTT assays were applied to assess the inhibitive effects of cisplatin on cell proliferation. The results showed that inhibition of FUNDC1 promoted the killing effect of cisplatin on HeLa and Caski cells (Fig. 3A). Under the action of ionizing radiation (2, 4, 6, and 8 Gy), the clonality of HeLa and Caski cells with stable FUNDC1 gene silencing was decreased significantly (Fig. 3B). This result suggests that downregulation of FUNDC1 increases radiosensitivity in cervical cancer cells. Moreover, after replenishment of FUNDC1 in both FUNDC1‐silenced HeLa and Caski cells, the altered chemoradiosensitivity was recovered (Fig. 3A and B).

### Inhibition of FUNDC1 promotes cisplatin and ionizing radiation‐induced DNA damage and apoptosis

**Figure 4:**
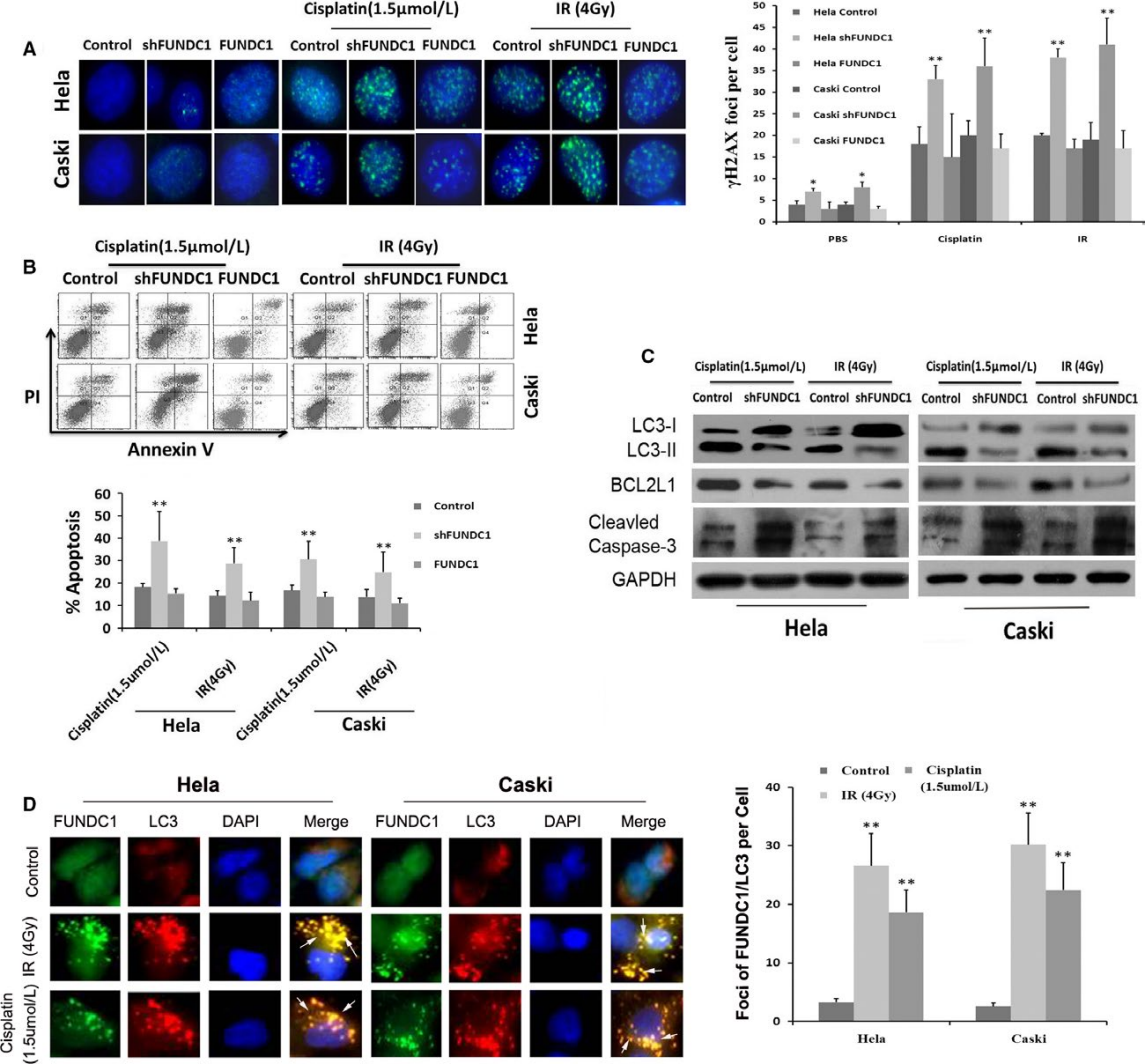
Depletion of FUNDC1 promotes cisplatin and IR‐induced DNA damage and apoptosis of cervical cancer cells. (A) Silencing of FUNDC1 inhibits the repair of cisplatin and IR‐induced DNA damage. Cells were subjected to cisplatin and IR at indicated dose, 12 h later, fixed for immunofluorescence. Shown is staining with antibodies to γH2AX (green). *γ*H2AX foci used as a measure of unrepaired DNA damage. (B) Knockdown of FUNDC1‐enhanced Cisplatin and IR‐induced cell apoptotic events in Hela229 and CaSKi cells monitored by Annexin V‐APC/PI staining and flow cytometry assays (the rate of cell apoptosis = D2% + D4%). (C) After treatment with Cisplatin or IR, LC3, BCL2L1, and cleaved Caspase‐3 were determined by Western blotting, and GAPDH was used as a normalized control. (D) Cells treated with IR or Cisplatin were visualized by immunofluorescence with anti‐FUNDC1 (green) and anti‐LC3 (red). Merged foci showed interaction between FUNDC1 and LC3. The average numbers of IR and Cisplatin‐induced foci of FUNDC1/LC3 per cell were quantified with Image J software. All data represent the mean ± SE derived from three individual experiments with triplicate wells. (*P <* *0.05, **P <* *0.01, Student's *t*‐test)

Both cisplatin and ionizing radiation induce double‐stranded DNA damage and apoptosis in tumor cells, thereby exerting antitumor effects. We further analyzed the effect of FUNDC1 inhibition on cisplatin and ionizing radiation‐induced DNA damage and apoptosis. Immunofluorescence was used to detect the formation of phosphorylated histone 2AX (γH2AX) foci and evaluate the extent of DNA damage. The results showed significantly more γH2AX foci in shFUNDC1 group cells after cisplatin and irradiation than in control group cells (Fig. 4A, **P <* *0.01). These data suggest that inhibition of FUNDC1 promotes DNA damage caused by cytotoxic drugs and radiotherapy. Annexin V‐APC/PI apoptosis assay results also suggested that FUNDC1 gene silencing in HeLa and Caski cells promoted the apoptosis caused by cisplatin and ionizing radiation (Fig. 4B, **P <* *0.01). On the other hand, reexpression of FUNDC1 in both FUNDC1‐silenced HeLa and Caski cells, the altered IR (ionizing radiation), and Cisplatin‐induced DNA damage and apoptosis were all recovered (Fig. 4A and B). Furthermore, depletion of FUNDC1 decreased expression of the antiapoptotic protein BCL2L1, and inhibited the activation of autophagic protein LC3 under the effects of cisplatin and ionizing radiation, which promoted activation of Caspase‐3 (Fig. 4C). Previous study has revealed that hypoxia treatment, which induces the colocalization and interaction between FUNDC1 and LC3‐II, results in trigging the activation of mitophagy 16. Interesting, our study showed that IR and Cisplatin treatment could also induce the colocalization and interaction between FUNDC1 and LC3‐II (Fig. 4D).

## Discussion

Through detecting FUNDC1 expression in the postoperative tumor tissue for 82 patients with early‐stage cervical cancer, we found significantly higher expression of FUNDC1 compared with that in normal cervical tissues. Furthermore, FUNDC1 protein expression could be used as an independent prognostic factor for patients with early‐stage cervical cancer, and it was negatively correlated with their OS and DFS. In addition, our results suggest that FUNDC1 promotes the proliferation of cervical cancer cells and inhibits apoptosis.

When mitochondria produce ATP and perform their related functions via the oxidation respiratory chain, these processes lead to the formation of ROS that mediate mitochondrial damage. By clearance of damaged mitochondria, mitophagy prevents further oxidative damage to cells, promotes cell survival, and inhibits apoptosis. In tumor incidence, development, and survival, mitophagy is thought to play a promoting role 17, 18, 19. However, the related studies have come to different conclusions. Both Parkin and BNIP3 promote mitophagy, but the lack of Parkin and BNIP3 in a mouse model promotes tumor incidence and development 20, 21. FUNDC1 is an important factor in newly discovered receptor‐dependent mitophagy, but its role in tumors remains unclear. Our data suggest that depletion of FUNDC1 inhibits the proliferation of Hela and Caski cervical cancer cells and promotes apoptosis. These results suggest that FUNDC1 may be a cancer‐promoting gene in cervical cancer. Parkin, BNIP3, and FUNDC1 promote mitophagy, but their roles in tumors are not the same. Studies suggest involvement of Parkin and BNIP3 in PTEN (phosphatase with tensin homology) and p53 tumor suppressor signaling pathways, but they only promote mitophagy under a particular stress 12, 18. The roles of these molecules in tumors may be mediated mainly by signaling pathways other than those in mitochondria. Mitochondrial membrane‐bound FUNDC1 protein, as a more dedicated mitophagy regulatory factor, can activate mitophagy in tumor cells may play a role as a cancer‐promoting gene 22.

All postoperative cervical cancer patients enrolled in this study had received adjuvant cisplatin‐based chemotherapy and radiotherapy. Therefore, we evaluated the effect of FUNDC1 on cervical cancer chemoradiotherapy sensitivity in vitro. Our results suggested that inhibition of FUNDC1 promoted the death of cervical cancer cells under the effects of cisplatin and ionizing radiation. Regardless of cytotoxic drugs or ionizing radiation, the main mechanism to kill tumors is cellular DNA and organelle damage that activates programmed cell death pathways by endogenous or exogenous pathways. The mitochondrial pathway is the main death signaling pathway 23, 24. Some important regulatory factors, such as p53, PI3K/Akt, and Bcl2‐Beclin‐1, jointly regulate apoptosis and autophagy processes, so that the two act synergistically 25, 26. Through savaging damaged organelles for useful metabolites, autophagy helps tumor cells to overcome energy and nutrition shortages, protect tumor cells from damage by cytotoxic drugs and ionizing radiation, and support cancer cells to resist apoptosis and maintain continued cell growth 27. Mitophagy also promotes cell survival by removing damaged mitochondria and ROS, and inhibiting activation of apoptosis signaling pathways.

As an integrated mitochondrial outer‐membrane protein, FUNDC1 can serve as a receptor for mitophagy caused by hypoxia. Herein, our study showed that IR and Cisplatin treatment could also induce the colocalization and interaction between FUNDC1 and LC3‐II, which may trigger the activation of mitophagy and clear damaged mitochondrial induced by IR and Cisplatin. These functions may further inhibit activation of the apoptosis pathway and promote tumor cell survival under stress and injury conditions. Our results also suggest that inhibition of FUNDC1 in cervical cancer cells promotes DNA damage and apoptosis pathway activation induced by cisplatin and ionizing radiation.

In this study, for the first time, we examined the FUNDC1 expression profile in tumor tissues of patients with cervical cancer, and found that high FUNDC1 expression can be used as an independent predictor for poor prognoses of patients. Furthermore, our study suggests that FUNDC1 has potential oncogene characteristics, and inhibition of FUNDC1 promotes the sensitivity of cancer cells to chemoradiotherapy. Therefore, FUNDC1 may become a new therapeutic target for cervical cancer and other tumors, and improve the prognosis of cancer patients.

## Conflict of Interest

The authors declare no financial support or conflict of interest.

## Supporting information


**Figure S1.** The mRNA expression pattern of FUNDC1 in cervical cancer and adjacent normal tissues.

**Data S1.** Materials and Methods.
