# Supplementary figures and images for: High expression of FUNDC1 predicts poor prognostic outcomes and is a promising target to improve chemoradiotherapy effects in patients with cervical cancer
Source: Cancer Med. 2017 Jul 18;6(8):1871–81. doi: 10.1002/cam4.1112 (PMC5548885; doi:10.1002/cam4.1112)

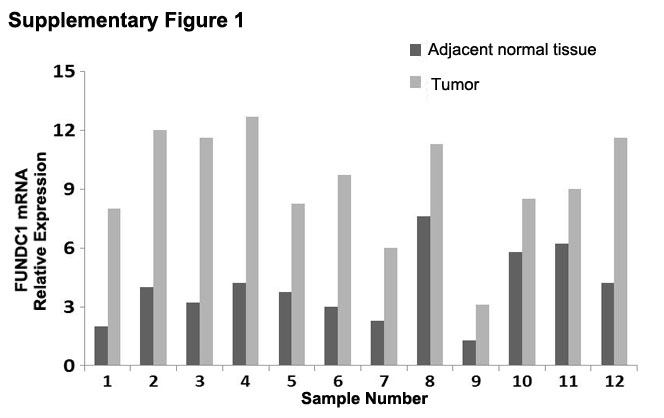

Supplement: Supplementary file 1 — Figure S1. The mRNA expression pattern of FUNDC1 in cervical cancer and adjacent normal tissues. [file CAM4-6-1871-s001.tif]
