# Supplementary material for: High expression of FUNDC1 predicts poor prognostic outcomes and is a promising target to improve chemoradiotherapy effects in patients with cervical cancer
Source: Cancer Med. 2017 Jul 18;6(8):1871–81. doi: 10.1002/cam4.1112 (PMC5548885; doi:10.1002/cam4.1112)
Supplement: Supplementary file 2 — Data S1. Materials and Methods. [file CAM4-6-1871-s002.docx]

**Supplementary Figure legends**

**Supplementary Figure 1： The mRNA expression pattern of FUNDC1 in cervical cancer and adjacent normal tissues.** FUNDC1 mRNA expression in human cervical cancer tissues (CCs) and adjacent normal tissues (ANTs) detected by Real-time PCR. FUNDC1 mRNA expression in 12 primary CCs is up-regulated compared to their ANTs.

**Supplementary Materials and Methods**

**RNA extraction, reverse transcription and real-time PCR**

Total RNA was isolated from 12 pairs of cervical cancer tissues (CCs) and their adjacent normal tissues (ANTs) using TRIZOL reagent (Invitrogen). The extracted RNA was pretreated with RNAase-free DNase, and 2ug RNA from each sample were used for cDNA synthesis primed with random hexamers. Real-time PCR was carried out using an ABI 7900HT fast real-time system (Applied Biosystems, Foster City, California, USA) to determine the expression pattern of FUNDC1mRNA in each of the CC sample as well as the paired ANT tissue. Expression data were normalized to the geometric mean of the housekeeping gene glyceraldehydes 3-phosphate dehydrogenase (GAPDH). The cDNA products were amplified with GAPDH-specific (F: 5’-CCACCCATGGCAAATTCCATGGCA-3’ and R: 5’- TCTAGACGGCAGGTCAGGTCCACC-3’) and FUNDC1-specific (F: 5’-ATGGGTGGCGTTACTGGC-3’ and R: 5’- TGCTTTGTTCGCTCGTTT -3’) primers by PCR.
